# Bovine Papillomavirus 1 Gets Out of the Flock: Detection in an Ovine Wart in Sicily

**DOI:** 10.3390/pathogens9060429

**Published:** 2020-05-30

**Authors:** Federica Savini, Laura Gallina, Alice Prosperi, Roberto Puleio, Antonio Lavazza, Patrizia Di Marco, Serena Tumino, Ana Moreno, Davide Lelli, Annalisa Guercio, Alessandra Scagliarini

**Affiliations:** 1 Dipartimento di Scienze Mediche Veterinarie, Università di Bologna, Ozzano Emilia, 40064 Bologna, Italy; laura.gallina@unibo.it (L.G.); alessand.scagliarini@unibo.it (A.S.); 2 Istituto Zooprofilattico Sperimentale della Lombardia e dell’Emilia Romagna “Bruno Ubertini”, 25124 Brescia, Italy; alice.prosperi@izsler.it (A.P.); antonio.lavazza@izsler.it (A.L.); anamaria.morenomartin@izsler.it (A.M.); davide.lelli@izsler.it (D.L.); 3 Istituto Zooprofilattico Sperimentale della Sicilia, 90129 Palermo, Italy; roberto.puleioizs@gmail.com (R.P.); patrizia.dimarco@izssicilia.it (P.D.M.); annalisa.guercio@izssicilia.it (A.G.); 4 Dipartimento agricoltura, alimentazione, ambiente, sezione Produzioni animali, Università di Catania, 00159 Catania, Italy; serena.tumino@unict.it

**Keywords:** papillomavirus, sheep, NGS, wart

## Abstract

A proliferative cauliflower lesion was excised from the udder of a sheep. Histological investigation confirmed the macroscopic classification of the lesion as a papilloma, without any fibroblastic proliferation. PCR revealed the presence of bovine papillomavirus (BPV), which was further confirmed by the identification of a *Deltapapillomavirus* *4* by Next Generation Sequencing analysis. This was subsequently classified as bovine papillomavirus type 1. Negative staining electron microscopy (EM) analyses produced negative test results for papillomavirus particles. RNA in situ hybridization (ISH) confirmed the presence of BPV-1. The results further confirm the ability of BPVs belonging to the *Deltapapillomavirus* genus to infect distantly related species and to cause lesions that are different from sarcoids.

## 1. Introduction

Papillomaviruses (PVs) are the only members of the family Papillomaviridae. Classification in genera, species and types is based on the most conserved L1 gene, which encodes for the major capsid protein. PVs’ nomenclature is related to the host in which they are first isolated, in agreement with the ICTV code [1], since they are considered highly species-specific viruses [1,2]. Among animals, bovine papillomaviruses (BPVs) are the most intensively studied, given their importance in comparative pathology [3]. To date, a total of 27 BPVs have been detected; three types still remain unclassified, while the others belong to five different genera (*Delta-*, *Xi-*, *Epsilon-*, *Dyoxi-* and *Dyokappapapillomavirus*) [4]. In particular, the *Deltapapillomavirus* genus comprises viruses infecting different animal species within the Bovidae, Cervidae and Giraffidae families. BPVs belonging to the *Deltapapillomavirus* genus are capable of causing sarcoids in distantly related hosts such as horses, mules, African lions [5], domestic cats [6], Cape mountain zebras, giraffes and sable antelopes [7,8]. Sarcoids are locally invasive fibroblastic skin tumors, histologically characterized by epidermal hyperplasia and sub-epidermal proliferation of ‘fibroblast-like’ cells. The BPV infection in distantly related hosts is considered abortive [9,10,11,12], meaning that the productive cycle of the virus is not complete, BPV exists episomally and no mature virions can be demonstrated or observed by electron microscopy.

Recent findings have demonstrated that sheep are also permissive hosts for BPVs. Among deltapapillomaviruses, BPV-2 has been detected in sheep warts from Brazil [13]; BPV-1, -2 and -13 have been detected in the blood of healthy sheep from Sardinia and Campania [14,15]; while BPV-2 and BPV-13 DNA and E5 oncoprotein have been documented to be expressed in congenital lesions on lambs in a flock in Sardinia, in the form of proliferative tissues in the gingiva and oral mucosa [15].

In this study, BPV-1 was detected in a proliferative lesion of a sheep, suggesting a novel trans-species infection and showing a different pathogenetic role of BPV-1, one that causes a nonsarcoid outcome on cutaneous surfaces.

## 2. Results

### 2.1. Histopathology

**Figure 1:**
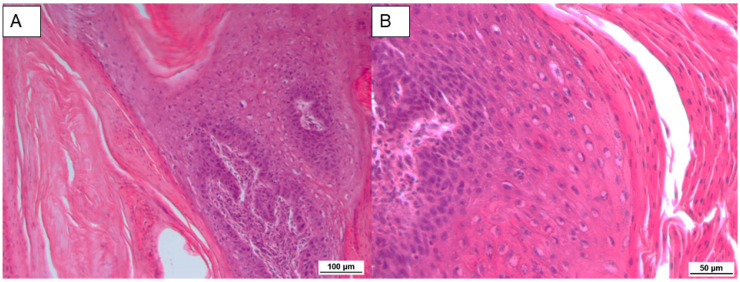
(**A**) Papillae with an irregular rete ridge formation covered by an acanthotic epidermis. Hematoxylin–eosin (HE) 10×. (**B**) Single and small groups of cells with vacuolated cytoplasm in the spinous layer. HE 20×.

Histopathologically, the tumor was composed of connective tissue, covered by a hyperplastic epidermis with acanthosis and marked ortho- and parakeratotic hyperkeratosis. The papillae were supported by a core of dermal fibrous connective tissue with irregular, elongated and expanded rete ridges (Figure 1A). In the spinous layer, some cells showed eccentric pyknotic nuclei, a perinuclear halo and vacuolated cytoplasm (koilocytes) with the presence of clumped and variably sized keratohyalin granules (Figure 1B). Occasionally, intranuclear inclusion bodies in the basal layer were observed.

### 2.2. In Situ Hybridization

**Figure 2:**
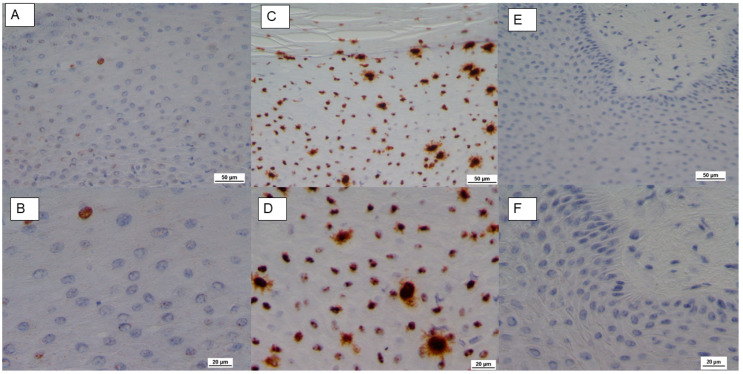
RNA in situ hybridization for bovine papillomavirus type 1 E5 gene (V-BPV-E) mRNA detection. (**A**,**B**) 20× and 40× respectively, showing the presence of rare clusters of brown dots in epithelial cells in sample IT-1506. (**C**,**D**) Positive control: 20× and 40× respectively. (**E**,**F**) Negative control: 20× and 40× respectively.

RNA in situ hybridization (ISH), used to detect E5 mRNA (V-BPV-E), revealed rare and intermittent clusters of brown dots in the epithelial cells (Figure 2A,B), as compared to positive (Figure 2C,D) and negative controls (Figure 2E,F).

### 2.3. Molecular Biology

PCR amplification of the ovine cytochrome C oxidase subunit I (COX I) gene demonstrated DNA viability, and the obtained sequence showed 100% nucleotide identity to the *Ovis aries* COX I gene. No parapoxvirus was detected by PCR, while the sequencing of the E5 gene of IT1506 demonstrated 100% nucleotide identity to the BPV-1 (GenBank accession number MG977494.1). The nucleotide sequence was submitted to GenBank with the accession number MT013334. The digestion of the rolling-circle amplification (RCA) product with *BamHI* and *HindIII* restriction enzymes generated a faint pattern. In particular, *BamHI* digestion produced two fragments of about 3 and 4.5 kb, while *HindIII* produced a single fragment of about 5 kb.

Illumina MiSeq produced 3,020,558 raw reads that were filtered for quality (using FastQC) and trimmed (using the FASTX Trimmer). The high-quality paired-end reads were de novo assembled into 1013 contigs, which were analyzed using BLASTn/BLASTx with the National Center for Biotechnology Information (NCBI) databases. One contig showed a 100% nucleotide identity to *Deltapapillomavirus 4* (GenBank accession number MF384293), and it was composed of 1587 reads (with a mean coverage of 43.7). The consensus sequence of the complete viral genome was obtained (7945 nucleotides) and was compared to the consensus sequence obtained from a mapping assembly against the BPV-1 sequence NC_001522. This consensus sequence was built from 1747 reads and showed a 100% nucleotide identity to the consensus sequence obtained from de novo assembling. The IT_1506 complete genome was submitted to GenBank with the accession number MT119079.

### 2.4. Electron Microscopy

No mature viral particles morphologically referable to papillomaviruses were observed by negative staining electron microscopy.

## 3. Discussion

The ability of BPVs to cause lesions in distantly related animal species was first described in 1951 [16], when horses that had been intradermally inoculated with a purified bovine wart extract displayed the development of transient sarcoid-like tumors. A deltapapillomavirus infection causes an initial transformation of the subepithelial fibroblasts, followed by epithelial plexiform acanthosis and subsequent papillomatosis [17].

In addition, BPV-1 and -2 have been documented in healthy and proliferative skin samples of wild and domesticated species [18,19,20].

The bovine deltapapillomaviruses have the biological property of being able to infect not only the epidermal but also the dermal cells, leading to the formation of fibropapillomas in cattle [21] and sarcoids in horses [22]. They are therefore commonly termed fibropapillomaviruses. In addition, Roperto and colleagues [14,15] recently demonstrated that BPV-2 and -13 can also cause fibropapillomas in sheep. Herein, we report evidence that BPV-1 is not only capable of infecting sheep, but is also able to cause papillomas and cutaneous lesions without fibroblastic involvement. Similar behavior has been previously described for BPV-2 in infected sheep in Brazil [13], where lesions identified as papillomas by histopathological analysis were localized on the hind feet.

NGS analysis resulted in the identification of BPV-1 as the unique viral agent present in the lesion, evidencing its role as the major etiological agent of the skin papilloma. Such a result was also supported by the detection of viral V-BPV-E mRNA by ISH. This technique, based on RNA detection, proved to be a robust method for the detection of high-risk human papillomaviruses (HPVs) and also proved to be more sensitive than DNA ISH in detecting HPV in oral squamous cell carcinomas [23,24,25].

The RNA ISH confirmed the presence of BPV, detecting the expression of the E5 gene, which encodes for an important small transmembrane oncoprotein required for cell transformation and known to contribute to oncogenic activities and tumor progression [26]. Our findings confirm the cytoplasmatic localization of the E5 oncogenic protein, which has been extensively investigated in the literature and shown to be within the cytoplasm of infected epithelial and fibroblastic cells and predominantly localized in the Golgi apparatus [27,28,29] 

Four PVs belonging to two different genera have been identified in sheep (*Ovis aries*). *Ovis aries* papillomavirus (OaPV) types 1, 2 and 4 are associated with cutaneous fibropapillomas, and OaPv-3 was isolated from a squamous cell carcinoma [29,30,31]. NGS did not detect any of these PVs. The IT-1506 genome sequence showed 100% identity with a BPV-1 isolated from a cow fibropapilloma in Switzerland. This result reinforces the concept that the genomic characteristics of BPV-1 are not related to the species from which they are isolated, as stated by Koch and co-workers [32]. Moreover, the fact that the source of the BPV-1 IT 1506 is a papilloma lesion and not a fibropapilloma led us to speculate that the outcome of the infection might be the result of virus––host interaction.

Papillomaviral transmission is thought to take place mainly via the horizontal route, but vertical transmission of human papillomavirus (HPV) in humans [33], as well as of BPV in horses [34] and sheep [15], has been demonstrated. In addition, transmission might also take place via contaminated material, habitual surroundings [35] and BPV-positive flies [36]. Contamination of grazing pasture by cattle suffering from chronic enzootic hematuria and BPV-associated bladder tumors that contain active BPVs has also been demonstrated [28]. Since the farmer reported sharing pasture between cattle and sheep, a contaminated environment could represent the source of the BPV infection. In addition, viral transmission between bovines and equids has also been described as a recurrent and ongoing phenomenon [32].

Whether the lesion is then able to be a source of infection itself remains to be investigated. Indeed, the negative results of the electron microscopy could be due to the relative sensitivity of the method, but in our experience, proliferative lesions on cattle do not always show overlapping PCR and electron microscopy (EM) results. Indeed, the absence of viral particles under EM is in agreement with previous results regarding interspecies infection. In fact, the so-called nonproductive infections, characterized by the absence of infective mature virions, have been described as the result of host jumps [37]. In these cases, bovine-associated papillomavirus DNA is consistently found, but papillomavirus particles cannot be evidenced because the virions may have a very low concentration [38]. Nevertheless, the presence of BPV-1 DNA in complex with the L1 capsid protein, defined as viral particle precursors, has been shown [39] in sarcoid samples, indicating that the virus could be productive at some stage of the viral replication cycle, despite the fact that intact virions are not detected. In our sample, NGS analysis revealed a low concentration of viral DNA (data not shown), supporting the hypothesis that, like equine sarcoids, lesions may contain infectious virions at low concentrations, or that BPV1/2 infections may be productive at least at some stages [22,38,39]. In this respect, the failure to detect viral particles by EM or by ISH could be due to the advanced development of the lesion.

Papillomaviruses are known to induce latency. The occurrence of sarcoids at sites of skin wounding and/or following physical trauma suggests a possible reactivation of BPV-1 and BPV-2 from latency [35,40], but other stimulating factors, such as mechanical irritation, wounding or UV irradiation, have been described as triggers. In particular, grazing on bracken fern is already known to be implicated in bladder-cancer induction in cattle in Sicily [41]. This factor might also be taken into consideration in our case, where a latent infection could have been triggered, leading to the formation of a papilloma instead of a sarcoid. Contagious ecthyma caused by the Orf virus (OV) in sheep mostly causes pustular dermatitis on muzzles and udders; in the atypical form, however, the infection causes extensive proliferative skin lesions, grossly resembling warts [42,43,44]. Furthermore, evidence of co-infections between poxviruses and papillomaviruses within the same lesion have been described [44,45].

None of the diagnostic methods employed (histology, electron microscopy and PCR) demonstrated the presence of parapoxvirus in the lesion, adding evidence that the causative agent of the proliferation was the BPV-1 IT-1506. This further supports the expansionist trends of deltapapillomaviruses.

## 4. Materials and Methods

### 4.1. Sample Collection

**Figure 3:**
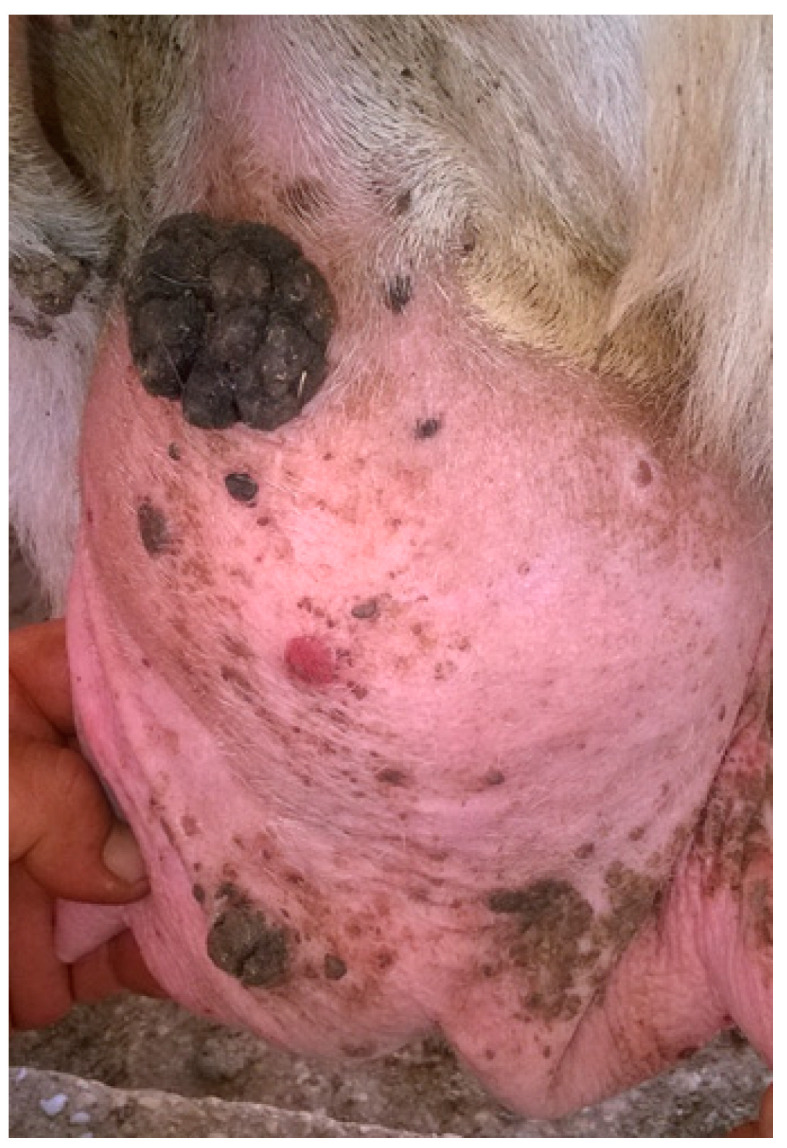
Macroscopic features of the analyzed sample: a large cauliflower lesion on the udder of a ewe.

A large (>10 cm) cutaneous lesion with a verrucous hyperkeratotic cauliflower-like aspect was excised from the udder of a ewe on a farm in Trapani province (Sicily) (Figure 3). Clinically, the lesion was classified as papilloma. This animal was farmed in a sheep holding, without any cattle reared in the same structure, even though a pasture shared by cattle and sheep was reported by the animal owner.

After the biopsy, the sample was divided into different fragments that were differently conserved and processed for diagnostic investigation using histology, electron microscopy (EM) and molecular biology.

### 4.2. Histological Evaluation

A skin sample, identified with the number IT-1506, was formalin-fixed and paraffin-embedded (FFPE), after which sections of 4 µm were hematoxylin–eosin (HE) stained and subsequently subjected to histological evaluation.

### 4.3. In Situ Hybridization

RNA in situ hybridization was performed using the RNAscope kit (Advanced Cell Diagnostics Inc., Hayward, CA, USA) according to the manufacturer’s instructions. The RNAscope probe used was RNAscope Probe-V-BPV-E (Catalog Number 416831), and was designed to detect the mRNA expression of the E5 gene (National Center for Biotechnology Information Reference Sequence NC_001522.1). FFPE tissue sections (of 5-µm thickness) were deparaffinized in xylene and were subsequently dehydrated in an ethanol series. Tissue sections were then incubated in a citrate buffer (10 nmol/L, pH 6), maintained at a boiling temperature (100 to 103 °C) using a hot plate for 15 min, rinsed in deionized water and immediately treated with Protease Plus at 40 °C for 30 min in a hybridization oven (Advanced Cell Diagnostics, Hayward, CA, USA). The tissue sections were then incubated at 40 °C with a target probe of BPV-1 in a hybridization buffer for 2 h. The target–probe hybridization was followed by a series of target-specific signal-amplification steps. After each hybridization step, slides were washed with wash buffer three times at room temperature. A horseradish-peroxidase-based signal-amplification system was then hybridized to the target probes, and this was followed by color development with 3,30-diaminobenzidine (DAB).

The sections were then counterstained with hematoxylin and dehydrated through ascending ethanol and xylene before being mounted with a mounting medium (Eukitt). The positive signals were present in the form of punctate cytoplasmic and nuclear brown staining that was higher than the signal on the negative control slide. Assays using FFPE specimens were performed in parallel with positive and negative control probes (positive probe Bt-PPIB; negative control probe-DapB), to ensure interpretable results.

### 4.4. Negative Staining Electron Microscopy

A frozen piece of the tissue sample was ground and homogenized (10% w/v in distilled water) before being examined by means of negative staining electron microscopy (NaPT 2%, pH 6.8) using the Airfuge method [46]. Samples were ultracentrifuged (Airfuge, Beckman Coulter Inc. Life Sciences, Indianapolis, IN, USA) for 15 min at 82,000× *g* by using a rotor that holds six 175-μL test tubes carrying specific adapters for 3 mm carbon-coated Formvar copper grids. The grids were then stained using 2% sodium phosphotungstate (NaPT), pH 6.8, for 1.5 min, and analyzed using a FEI Tecnai G2 Spirit BioTwin transmission electron microscope (FEI, Hillsboro, Oregon, USA) operating at 85 kV. Observations were made at (20,500 × *g* to 43,000× *g* for not less than 15 min before being declared negative. Identification of the observed viral particles was based on their morphological features.

### 4.5. DNA Amplification and PCR

DNA extraction with a NucleoSpin Tissue kit (MN) was performed on frozen tissue samples following the manufacturer’s instructions. DNA integrity was checked by amplifying a fragment of the *Ovis aries* COXI gene [44]. DNA was amplified by PCRs targeting the E5 ORF of BPV-1 and BPV-2 [22]. The diagnostic PCR amplification for parapoxvirus is based on the sequence of the B2L gene of ORFV, which is a homologue of the vaccinia virus major envelope antigen p37K [47]. All the PCR products were Sanger sequenced.

### 4.6. RCA

DNA was amplified by multiple-primed rolling-circle amplification (RCA) using the Illustra TempliPhi 100 amplification kit (GE Healthcare, Little Chalfont, UK) in agreement with the protocol indicated by Rector and colleagues [48]. Then, restriction enzymes *BamHI* and *HindIII* were used for performing digestion. The obtained products were run on a 0.8% agarose gel to confirm the presence of DNA fragments consistent with the length of a papillomaviral genome.

### 4.7. NGS Analysis

RCA DNA was prepared for NGS using the Nextera DNA Library Prep Kit (Illumina) according to the manufacturer’s instructions. Both DNA library concentration and quality were evaluated using the Qubit High Sensitivity dsDNA kit (Life Technologies) and the Agilent DNA High Sensitivity chip assay (Bio-Fab Research s.r.l, Rome, Italy). The library was sequenced on the MiSeq Platform (Illumina) using the MiSeq Reagent Kit v3 (2 × 300 cycles). The data were de novo assembled using SPAdes Genome Assembler v3.6 [14]. Assembled contigs were visualized using Geneious software (v10.2.6), and a consensus sequence was obtained and compared to the consensus sequence obtained by the mapping onto a reference assembly that was performed by Bowtie 2 (CodonCode v9.0.1).
